# Plasma sodium levels are related to resting motor threshold in healthy humans

**DOI:** 10.1038/s41598-025-28007-4

**Published:** 2025-12-18

**Authors:** Tamás Faludi, Ehssan Amini, Delia Christ, Christiane Gerhards, Elia Müggler, Annette Harings-Kaim, Thomas Schlitt, Andreas Papassotiropoulos, Dominique J.-F. de Quervain, Nathalie S. Schicktanz

**Affiliations:** 1 Division of Cognitive Neuroscience, Department of Biomedicine, University of Basel, 4055 Basel, Switzerland; 2 Research Cluster Molecular and Cognitive Neurosciences, Department of Biomedicine, University of Basel, 4055 Basel, Switzerland; 3 Psychiatric University Clinics, University of Basel, 4055 Basel, Switzerland; 4 Division of Molecular Neuroscience, Department of Biomedicine, University of Basel, 4055 Basel, Switzerland

**Keywords:** Calcium channels, Chloride channels, Potassium channels, Sodium channels, Epilepsy, Predictive markers, Ion channels in the nervous system, Neuroscience, Excitability

## Abstract

Electrolyte homeostasis is essential for normal neuronal function and clinically relevant imbalances can provoke neurological symptoms, including seizures. However, whether electrolyte variations within the normal physiological range affect cortical excitability remains unclear. In this exploratory secondary analysis, we examined baseline data from 42 healthy participants enrolled in a previously conducted clinical trial. We found a significant correlation between plasma sodium levels within the normal physiological range (136–143 mmol/L) and resting motor threshold (*r* = 0.47, *p* = 0.002), an indirect index of cortical excitability, suggesting that lower sodium concentrations may be associated with increased cortical excitability. No significant associations were observed for chloride, potassium, phosphate, or calcium. These preliminary findings raise the possibility that subtle interindividual differences in plasma sodium levels are related to variability in corticospinal excitability. Given that this was a non-prespecified, secondary analysis, further controlled studies are warranted to confirm the association and investigate the underlying mechanisms.

## Objective

Electrolyte homeostasis is critical for normal neuronal function, and disturbances in ion gradients can alter neuronal activity^1^. Clinically significant electrolyte imbalance such as hyponatremia (< 135 mmol/L) can precipitate neurological symptoms, including seizures, via multiple mechanisms^2–6^. Beyond hyponatremia, other electrolyte abnormalities (e.g., hypocalcaemia, hypomagnesemia) are associated with seizures^6,7^. These disturbances may influence excitability by altering the balance of key ions—including potassium and chloride—and are closely linked to sodium dynamics^8,9^. Despite these links under pathological conditions, whether physiological variation in sodium and other ions relates to cortical excitability remains unexplored.

Resting motor threshold (RMT), assessed via transcranial magnetic stimulation (TMS), serves as an indirect measure of cortical excitability at both cortical and spinal levels. Voltage-gated sodium channel (VGSC) blockers, a primary pharmacological treatment for epilepsy, have been shown to increase the motor threshold, i.e., the corticospinal system becomes less excitable^9^. RMT is usually defined as the minimum TMS intensity required to produce a motor-evoked potential (MEP) on 50% of pulses^10^. Although RMT varies between individuals, it demonstrates high test-retest reliability and low intra-individual variability^11^, suggesting that stable neurobiological characteristics determine RMT. Differences in electrolyte concentrations within normal physiological ranges may contribute to inter-individual variability.

Here, we tested whether plasma electrolyte levels within the normal physiological range are associated with RMT in healthy adults. We analysed baseline screening data from 42 healthy participants enrolled in a randomized clinical trial on fampridine and working memory^12^. This was a non-predefined, exploratory analysis.

## Methods

### Participants

From a clinical trial investigating the influence of fampridine on working memory in healthy young adults^12^ (ClinicalTrials.gov NCT04652557), we used plasma electrolyte values and RMT data collected at screening for the current analyses, resulting in *N* = 42 participants. All data assessed at screening were collected before initiation of any drug treatment. The entire study was performed in accordance with the Declaration of Helsinki. All participants gave written informed consent to trial participation. The manuscript contains only anonymized, aggregated data and does not include any identifying information or images. The study protocol was approved by the Swiss Ethics Committee (BASEC-ID 2020–02828), Swiss Agency for Therapeutic Products (Swissmedic), and pre-registered with ClinicalTrials.gov NCT04652557 (24-11-2020).

### Laboratory measurements

Blood samples were collected at the Department of Clinical Research of the University Hospital of Basel during the screening visit to assess inclusion criteria. All analyses were performed at the central laboratory of the hospital. Blood was drawn using 2.7 ml EDTA and 4.7 ml Li-Heparin Monovettes. The following parameters were measured: complete blood count (including platelets, haemoglobin, and haematocrit) and blood chemistry (including sodium, potassium, chloride, calcium, phosphate, creatinine, uric acid, bilirubin, alkaline phosphatase, LDH, AST, ALT, GGT, pancreatic amylase, albumin, total protein, CRP, and creatine kinase).

### RMT assessment

RMT was defined as the lowest stimulation intensity that induced an MEP of ≥ 50 µV peak-to-peak amplitude in the relaxed abductor digiti minimi of the dominant hand in at least 5 out of 10 trials. TMS was performed using a biphasic Magstim Rapid2 stimulator (The MAGSTIM^®^ Company Ltd, Whitland, UK) and a 70 mm figure-of-eight coil. To locate the hotspot, the coil was positioned tangentially to the skull and aligned over the estimated area representing the abductor digiti minimi muscle in the primary motor cortex. We used the standard brain template in the Brainsight neuronavigation system. The coil handle was angled 45° backward and lateral to the parasagittal line.

Stimulation began at 30% of maximum stimulator output (MSO), with pulses applied to the initial point and four surrounding points spaced 1 cm apart. Intensity was gradually increased in 10% steps until an MEP was detected. In cases of uncertainty, intensity was increased again to confirm the location. Then, RMT was assessed. Ten consecutive pulses were applied at this location with 5–8 s intervals. If 5 or more MEPs were detected within the 10 pulses, intensity was reduced by 5%; otherwise, it was increased by 5%. This adjustment continued in 2% and then 1% increments until the lowest intensity that produced at least 5 MEPs was found, defining the RMT. To confirm, intensity was reduced by 1% at the end. EMG recordings were acquired using the built-in two-channel EMG device of the Brainsight system. Surface Kendall Foam electrodes were placed in a belly-tendon montage over the abductor digiti minimi muscle, with a ground electrode on the dorsal wrist near the ulnar styloid process.

### Statistical analysis

All statistical analyses were conducted in R (version 4.3.2)^13^ using robust non-parametric regression models (Rfit) due to violations of residual normality in linear models. The dependent variable was RMT, with blood electrolytes as independent variables and sex and age as covariates. Effect sizes (r) were calculated from model t-values. Bonferroni correction was applied to account for five independent tests.

## Results

Age ranged from 18 to 30 years (mean = 24.12, sd = 3.10) and the sample consisted of 23 females and 19 males. For baseline characteristics regarding blood values and RMT refer to Table 1.

**Table 1:** Descriptives of blood baseline and RMT values.

	Mean	Median	SD	SE	Q25	Q75	min	max
RMT (%)	64.98	62	12.9	1.99	55	73.5	47	95
Sodium (mmol/L)	139.36	140.00	1.86	0.29	138.00	140.75	136.00	143.00
Chloride (mmol/L)	103.95	104.00	2.34	0.36	102.25	106.00	99.00	108.00
Potassium (mmol/L)	3.95	3.90	0.23	0.04	3.80	4.10	3.60	4.40
Phosphate (mmol/L)	1.07	1.09	0.14	0.02	0.99	1.16	0.71	1.37
Calcium (mmol/L)	2.34	2.34	0.09	0.01	2.27	2.40	2.15	2.57

*SD* standard deviation, *SE* standard error of the mean, *Q25* 25th percentile (lower quartile), *Q75* 75th percentile (upper quartile), *min* minimum observed value; max = maximum observed value.

**Fig. 1:**
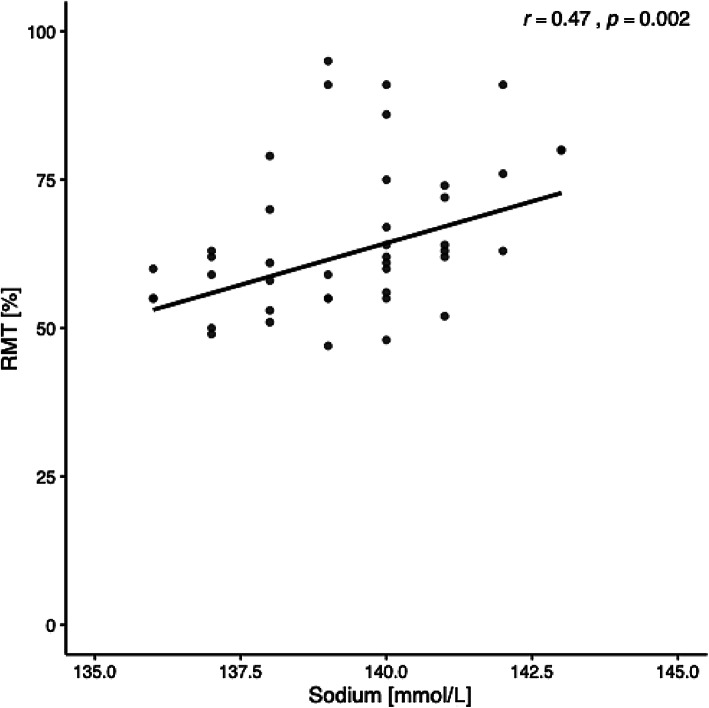
Correlation between plasma sodium (mmol/L) levels and resting motor threshold (RMT) (%).

Lower blood sodium levels were significantly associated with lower RMT, suggesting increased cortical excitability (*t*(38) = 3.29, *p* = 0.002, *pBonf* = 0.011, *r* = 0.47, see Fig. 1). Bonferroni correction was applied to the exact p value from the statistical test before rounding. Chloride (*t*(38) = 1.59, *p* = 0.12, *r* = 0.25), potassium (*t*(38) = 1.47, *p* = 0.15, *r* = 0.23), calcium *t*(38) = − 1.4, *p* = 0.17, *r* = − 0.22), and phosphate (*t*(38) = − 1.31, *p* = 0.20, *r* = − 0.21) levels did not show significant associations with RMT (see Table 2). Across all five models, no significant three-way interactions between age, sex, and electrolyte were found (all *p* > 0.07), nor any two-way interactions (all *p* > 0.05) or main effects of age or sex (all *p* > 0.23) on RMT.

To further explore the potential role of calcium in cortical excitability, we examined albumin-corrected calcium levels provided by the clinical laboratory, which are commonly used in clinical settings as a pragmatic surrogate for ionized calcium. A slightly stronger, though non-significant, correlation was found between RMT and albumin-corrected calcium levels (*t*(38) = − 1.86, *p* = 0.07, *r* = − 0.29) compared with total calcium levels.

In a separate model with sodium as the dependent variable, no significant main effects of age, sex, or their interaction were found (all *p* > 0.51).

**Table 2:** Association between RMT and blood values sorted descending based on effect size.

Electrolyte	Estimate (B)	SE	*p*	*r*
Sodium	2.81	0.85	0.002	0.47
Chloride	1.37	0.86	0.120	0.25
Potassium	13.14	8.93	0.149	0.23
Phosphate	− 18.75	14.30	0.198	− 0.21
Calcium	− 31.96	22.79	0.169	− 0.22

Table 2 is showing robust non-parametric regression models, with RMT as the dependent variable and each electrolyte entered separately as a predictor, controlling for age and sex. Effect sizes (*r*) were calculated from model *t* values using the formula: √(t² / (t² + df)). Negative signs were retained to indicate direction of association. SE = standard error; *p* = p value; *r* = effect size; B = unstandardized regression coefficient.

### Interpretation

This study aimed to investigate the correlation between plasma electrolytes in the physiological range and RMT. Our results indicate a positive correlation between plasma sodium levels and RMT. RMT is influenced by central and peripheral factors—including spinal motoneuron excitability, neuromuscular transmission, and anatomical electric-field dosing^11^. Nevertheless, because RMT is widely used as an indirect index of corticospinal excitability^9,14^, we interpret the sodium–RMT association as consistent with higher excitability, while refraining from causal inference. It is important to note that we cannot make any inferences about the impact of variations of sodium levels in the physiological range and seizure risk.

The excitability of neurons is characterized by membrane properties, the membrane potential and ion concentrations in- and outside of the cell^1,15^. There are several mechanisms by which small differences in plasma electrolyte levels could influence the excitability of cortical neurons. In terms of sodium, a key biological explanation is that lower extracellular sodium concentrations may enhance neuronal excitability by modulating the biophysical properties of neuronal membranes. Among plasma electrolytes, extracellular sodium has a particular role in shaping membrane excitability. A reduction in extracellular sodium ([Na⁺]ₒ) across our observed physiological range (136–143 mmol/L) changes the sodium equilibrium potential (Eₙₐ) by only ~ 1–2 mV^16,17^. While this shift in driving force is modest, cortical and network excitability operate close to threshold, where even small changes can influence action potential initiation. Active and passive transport across the neuronal membrane regulates the resting membrane potential^18^. A modest decrease in [Na⁺]ₒ or membrane Na permeability is expected—per the Goldman–Hodgkin–Katz relation—to slightly hyperpolarize neurons even in neurons with small voltage-gated sodium channel (VGSC) currents at resting membrane potential, thereby reducing the likelihood of action potential generation^19,20^. However, such slight hyperpolarization may promote the recovery of voltage-gated sodium channels from inactivation, resulting in a more negative action potential threshold. This, in turn, could facilitate firing in response to weaker depolarizing stimuli^20^. Alterations in the extracellular ionic milieu can also affect voltage-gated sodium channel (VGSC) activation and inactivation, potentially lowering the effective spike threshold at the axon initial segment^16,17,21,22^. The resulting effects on action potential amplitude and width depend on the interplay with K⁺ conductance and inactivation kinetics; experimental work shows that altering [Na⁺]ₒ can change firing rates and action potential morphology^23,24^, though reported directions vary with preparation and range. In the MEP/RMT context, postsynaptic AMPAR currents are minimally affected by a ~ 1 mV Eₙₐ shift, but pre-synaptic Na⁺-dependent Ca²⁺ handling and osmolarity-driven extracellular space changes (affecting K⁺ accumulation and ephaptic coupling) can enhance effective synaptic transmission^15,23^. Evidence from experimental studies supports these mechanisms: in cultured hippocampal neurons, lowering [Na⁺]ₒ within the physiological range increased firing rates and decreased action potential width^23^. Computational simulations informed by sodium MRI data further suggest that even modest shifts in sodium concentrations (e.g., 145–160 mmol/L) can influence neuronal activity patterns^25^. Network models further suggest that even subtle alterations in [Na⁺]ₒ can modulate the transitions between resting state, tonic activity, and seizure dynamics^26^. Together, these neuronal and network mechanisms provide plausible pathways by which lower [Na⁺]ₒ within the normal range could be associated with lower RMT (higher excitability). Nevertheless, RMT in our sample varied widely between individuals, and the proportion of this variability attributable to sodium concentration is unknown. Modelling studies suggest neuronal activity is sensitive to [Na⁺]ₒ, although prior simulations typically examined broader ranges than those in our study^25,26^.

Alternatively, the observed correlation might not directly reflect changes in cortical excitability but might be explained by physical changes in tissue conductivity. Because the intracranial electric field induced by TMS depends on the conductivity distribution of head compartments—including non-neuronal tissues (scalp, skull, CSF) as well as grey and white matter—variation in extracellular ionic composition can modulate electric-field strength for a given stimulator output. Sodium—the dominant extracellular cation—contributes substantially to extracellular conductivity; thus, higher Na⁺ could slightly increase conductivity and strengthen the induced electric field, lowering the strength for a given stimulator output required to reach motor threshold without any change in neuronal membrane properties^27,28^. The magnitude of any such effect from physiological sodium variability (136–143 mmol/L in our sample) is unknown and remains to be quantified. Notably, in vitro studies show that altering extracellular Na⁺ changes neuronal firing and action-potential properties in the absence of external fields, supporting the plausibility of a direct Na⁺-dependent modulation of excitability^23^. On the recording side, Na⁺ could in principle influence EMG signal quality via the electrode–skin interface. Our data cannot separate these physiological and biophysical contributions, which may also co-occur. Future studies combining controlled sodium manipulations with individualized head modelling and electric-field simulations and/or conductivity imaging and impedance measurements will be needed to adjudicate between mechanisms. Furthermore, we did not estimate individual intracranial electric-fields, nor did we measure peripheral neuromuscular excitability; thus, non-cortical contributions to RMT cannot be excluded.

Because validated iCa-estimating equations typically require total CO₂ (and often pH) alongside albumin and phosphate, we could not compute estimated iCa retrospectively. Albumin-corrected calcium—a pragmatic proxy—showed a slightly stronger (non-significant) association with RMT than total calcium. Given known limitations of correction formulas without acid–base data, future studies should prioritize direct ionized calcium measurement and/or collection of the full panel needed to apply validated equations^29^.

For plasma sodium concentration to influence cortical activity, a key assumption is that plasma electrolyte levels correlate with ion concentration in the CSF and brain tissue. Sodium in the brain ultimately comes from the periphery; however, its uptake into the brain is controlled by active transport at the blood-brain-barrier (BBB) and blood-cerebrospinal fluid (CSF) barrier (BCSFB)^30^. Nevertheless, sodium may additionally be able to cross the BCSFB and BBB by diffusion^31^. Acute hyponatremia has repeatedly been associated with epileptic seizures, characterized by hyperexcitability of cortical neurons^3,5,8,15,32–34^, suggesting that plasma electrolyte levels to some extent impact electrolyte concentrations in the ECS. Even though acute drops in sodium have been primarily reported to increase seizure risk, also subacute hyponatremia has been found related to increased risk of epileptic seizures^35^.

The observed correlation between RMT and sodium concentrations, but not other electrolytes in the present study, is in line with the idea that plasma sodium concentrations are considered the main determinant of extracellular osmolarity^1,33^. Alternatively, the mechanism could also be indirect, as blood sodium concentration is regulated by the renin-angiotensin-aldosterone system (RAAS)^36^, and aldosterone itself can influence the CNS and affect cortical excitability^37^.

Given the observational, secondary, and non-prespecified design, we refrain from causal inference. The sodium–RMT relationship should be viewed as hypothesis-generating. Prospective studies with direct ion/acid–base measurements and TMS electric-field control are needed to evaluate causality and mechanisms.

## Data Availability

The data that support the findings of this controlled trial are not openly available due to reasons of participant privacy and are available from the corresponding author upon reasonable request. Data are located in controlled access data storage at the University of Basel.
